# Severe aberrant glenohumeral motor patterns in a young female rower: A case report

**DOI:** 10.1186/1746-1340-15-17

**Published:** 2007-11-13

**Authors:** Timothy W Stark, Jessica Seebauer, Bruce Walker, Neal McGurk, Jeff Cooley

**Affiliations:** 1 Health Science Division, Murdoch University, Murdoch, Western Australia, Australia

## Abstract

**Background:**

This case features an 18-year-old female with glenohumeral dysrhythmia and subluxation-relocation patterns. This unusual case highlights the need for careful examination and consideration to the anatomical structures involved.

Conventional approaches to shoulder examination include range of motion, orthopaedic tests and manual resistance tests. We also assessed the patient's cognitive ability to coordinate muscle function. With this type of assessment we found that co-contraction of local muscle groups seemed to initially improve the patients abnormal shoulder motion. With this information a rehabilitation method was instituted with a goal to maintain the improvement.

**Case presentation:**

An 18-year-old female with no history of trauma, presented with painless kinesiopathology of the left shoulder (in abduction) consisting of dysrhythmia of the glenohumeral joint and early lateral rotation of the scapula. Examination also showed associated muscle atrophy of the lower trapezius and surrounding general muscle weakness. We used an untested functional assessment method in addition to more conventional methods.

Exercise rehabilitation interventions were subsequently prescribed and graduated in accordance with what is known as the General Physical Rehabilitation Pyramid.

**Conclusion:**

This paper presents an unusual case of aberrant shoulder movement. It highlights the need for careful examination and thought regarding the anatomical structures and normal motor patterns associated with the manoeuvre being tested. It also emphasised the use of co-contraction during examination in an attempt to immediately improve a regional dysrythmia if there is suspicion of a regional aberrant motor pattern. Further research may be warranted to test this approach.

## Background and Methods

This case reports findings in an 18-year-old female who presented with motion aberration (kinesiopathology) of the left shoulder consisting of dysrhythmia of the glenohumeral joint and early lateral rotation of the scapula. To ascertain what is known about this type of condition a literature search was conducted via the database PubMed using the keywords "nontraumatic glenohumeral", "scapulohumeral", "subscapularis", "motor engram", and "glenohumeral instability", with the limits: All Adult (19+ years), English, Clinical Trial, Meta-Analysis, Practice Guideline, Randomised Controlled Trial, Review, Case Reports, Humans, Core Clinical Journals. This search did not return any case studies or clinical trials relating to this type of shoulder dysfunction, but several articles discussing shoulder kinematics and rehabilitation were located.

Glenohumeral joint stability is primarily dependant on muscle, hence it is often referred to as a muscular joint [1]. It is the most mobile joint in the human body, but the sacrifice for this mobility is stability, with glenohumeral instability (and resulting dysfunction) being a common finding [2]. While this anatomical arrangement predisposes the shoulder to traumatic changes, it is important to remember that non-traumatic dysfunction can also occur. Some cases relating to muscular imbalance of the glenohumeral and scapulothoracic joints [3] and faulty motor patterns (or joint region coordination) [4] have been reported. However none of these presented with findings similar to the case being reported. It is worth considering similar cases since many patients who present with shoulder pain continue to report pain 6–12 months later in spite of treatment [5].

Matias, et al [6] found that the faulty scapular kinematics of shoulder instability are perhaps related to suboptimal muscular activity. For example Bak [3], Blaimont, et al [7], Kuechle, et al [8], and Decker [9] all highlight the role the subscapularis plays in shoulder instability. Other important stabilizer functions of the shoulder region have been identified, including normal facilitation of the rotator cuff muscles and normal tone of the pectoralis major and deltoid muscles [10]. Labriola [10] states that if the pectoralis major and deltoid muscles are hypertonic, they also may promote glenohumeral instability.

Current trends in shoulder rehabilitation are numerous, varying from scapula-based [11] to kinetic chain [12] to more neuromotor-based [13] processes. Concurrently, shoulder rehabilitation protocols have been directed towards specific shoulder complex conditions, to include post-surgical [14], rotator cuff injury [15], and impingement [16]. It is claimed that inappropriate clinical attention to specific stabilizers, such as those that control the scapula, may result in further altered mechanics of the shoulder complex [17].

Stark [18] proposed a general physical rehabilitation pyramid guide as a theoretical construct. The base of this pyramid (Tier 1) is formed by a focus on cognitive facilitation, static proprioception (posture), and faulty mechanics correction. If motor coordination is learned and other aberrant conditions are "corrected", the patient is advised to progress through ascending levels of cardiovascular conditioning and dynamic proprioception training (Tier 2), stabiliser conditioning (Tier 3), mobiliser conditioning (Tier 4), and ADL (activities of daily living) conditioning (Tier 5).

This study reports the history, clinical examination, imaging and the challenging choice of management of a patient with an unusual shoulder movement dysfunction.

## Case presentation

### History

An 18-year-old athletically active female university student presented to the Murdoch University Chiropractic Clinic complaining of bilateral upper trapezius pain. She commented that she thinks she is "double jointed" because her shoulder "pops in and out of joint". The patient stated that she had had this shoulder dysfunction as long as she could remember. It had never caused her pain or limited her activities of daily living (ADL's), including rowing and playing stringed instruments; however she would prefer to not have the dysfunction. She denied any shoulder trauma or knowledge of any personal or family history of connective tissue disorders. Relevant medical history included a 20°C scoliotic curve at 11-years-of-age which progressed to 30°C in six months; after being braced for 1.5 years the curve decreased to 24°C and stabilised.

### Examination

The patient did not exhibit antalgia, and there was no obvious deformity of the left shoulder apparent upon static observation. Appearance and temperature of the skin about the neck, shoulders, and thoracic region were unremarkable, but generalised muscle tone and bulk at the left shoulder were subjectively decreased when compared to the right. Active range of motion (AROM) and passive range of motion (PROM) of both shoulders were full and pain-free in all directions. However, it was noted that the patient's left shoulder appeared to subluxate (or dislocate) and relocate from the glenoid fossa regularly between 75°C and 180°C of abduction. This dislocation/relocation pattern also appeared to occur to a lesser degree during flexion of the left shoulder. Manual muscle testing of the shoulder musculature revealed a mild weakness of the left supraspinatus (4/5). The neurological examination was unremarkable. Impingement tests were negative for pain, but excessive internal rotation of the left shoulder was demonstrated during Hawkins-Kennedy test [19] when compared to the right. Anterior instability tests were also pain-free, but positive for laxity on the left. A chiropractic examination did not reveal any suggestion of manipulable lesions in the shoulder complex.

### Functional examination

Observation of scapulohumeral rhythm revealed early lateral rotation of the left scapula, possibly due to chronic aberrant motor patterns including an early facilitation of the trapezius musculature and delayed serratus anterior and lower trapezius muscles. As a result, the normally smooth arc of shoulder abduction between 75°C and 180°C was punctuated by sharp, clunking, jerking movements which appeared to be due to the humerus repeatedly slipping from the glenoid fossa (see Additional file 1). The patient was instructed to co-contract her shoulder during abduction. This involved training the patient to contract the pectoralis minor, serratus anterior, subscapularis, latissimus dorsi and lower trapezius muscles. This complicated manoeuvre was facilitated by the clinician lightly pinching the posterior axilla muscle groups (latissimus dorsi and lower trapezius) by placing her fingers in the axilla from behind and having her thumb on the posterior aspect of the lower trapezius muscle, and then asking the patient to "contract these muscles". While co-contracting this muscle group and instructing the patient to perform the abduction movement, it was noted that the aberrant glenohumeral rhythm did not occur until the end range of the movement. There were also fewer episodes of glenohumeral clunking, allowing the patient to achieve a smoother arc of movement (see Additional file 2. Note: the patient performs abduction on the right demonstrating a normal movement pattern. She then performs an abduction manoeuvre on the left; first without co-contraction, then a second time with co-contraction).

### Radiological examination

**Figure 1:**
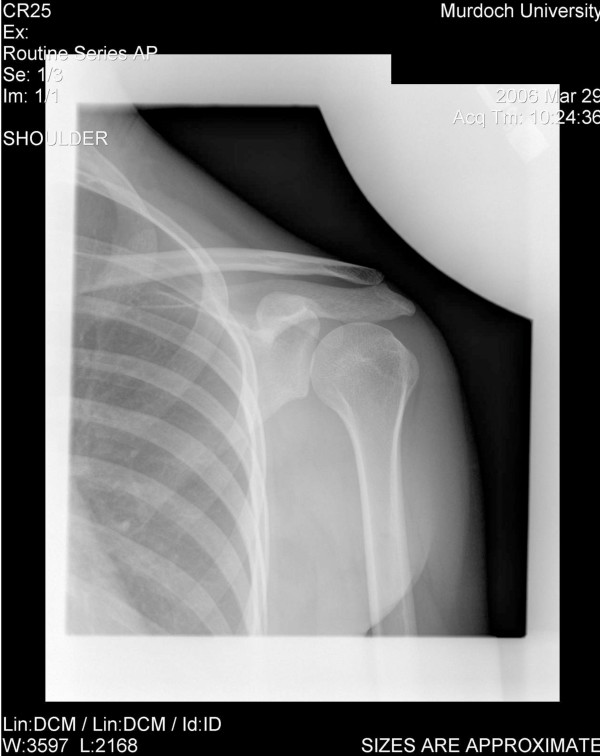
see attached jpeg file named "XRay 1".

**Figure 2:**
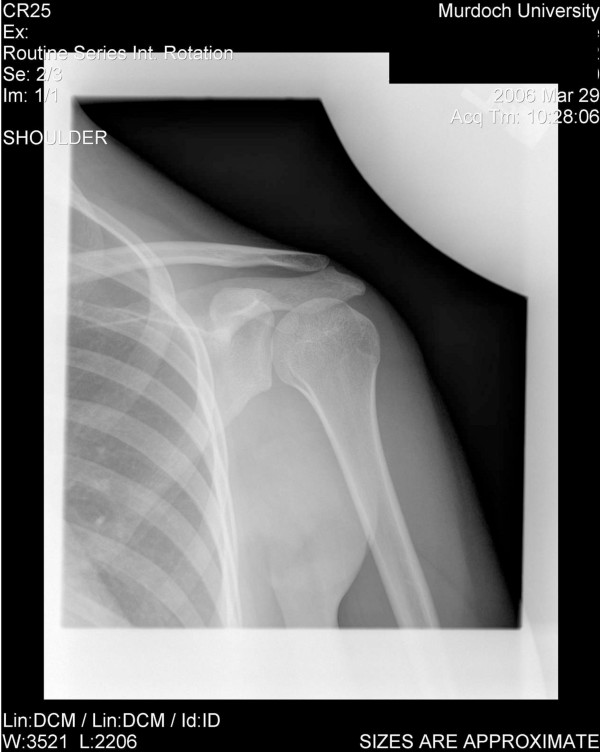
see attached jpeg file named "XRay 2".

**Figure 3:**
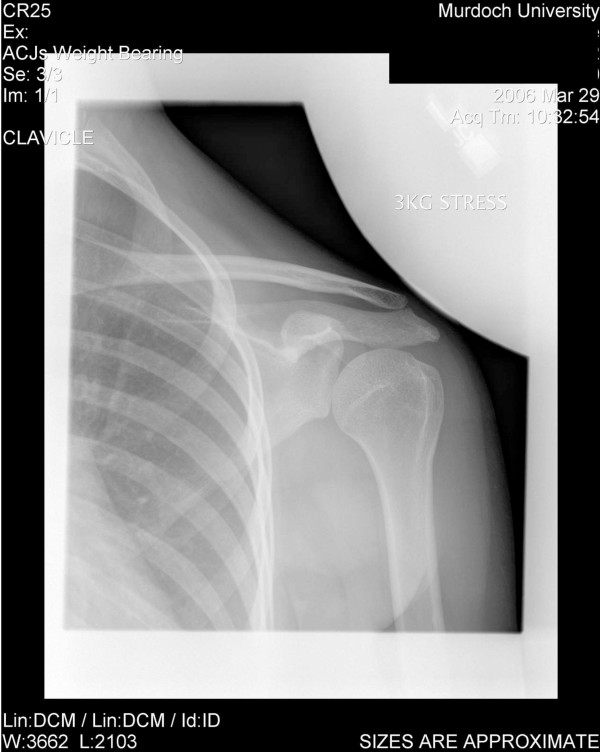
see attached jpeg file named "XRay 3".

Radiological investigation was ordered to rule out an anatomical aetiology (such as shoulder joint dysplasia) and confirm or deny an aberrant motor pattern as the sole cause of dysfunction. This consisted of plain film radiography and video fluoroscopy. A left shoulder series consisting of AP internal rotation, AP external rotation, and AP weighted (3 kg) neutral views (all taken in Grashey position [20]) revealed no bony dysplasia (Figures 1, 2, 3). Video fluoroscopy consisting of AP and axial projections confirmed the suspicion that the humerus subluxated inferiorly at the glenohumeral joint as it moved through the abduction arc. The axial projection showed a significant posterior component to this subluxation. A follow-up projection AP projection with co-contraction of the shoulder showed that these newly combined motor patterns kept the glenohumeral joint stable, making the arc of motion smoother, and reducing the dynamic subluxation. When viewing the following videos note the significant dysrhythmia for 4 repetitions followed by a smoother rhythm from the patient's conscious facilitation of co-contraction (see Additional file 3).

### Clinical diagnosis

Chronic, severe, non-traumatic, multidirectional instability of the left glenohumeral joint secondary to ligamentous laxity. This was accompanied by glenohumeral kinesio-pathology and aberrant scapulohumeral rhythm due to suboptimal motor patterns.

We opine that this unusual presentation was associated with facilitation of the upper trapezius with suspected inhibition of the subscapularis, lower trapezius, latissimus dorsi, serratus anterior and possibly the remaining rotator cuff. Without further EMG studies, this is simply the author's clinical opinion.

### Treatment Plan

Given the unusual presentation of this case, choice of therapy was problematic. The patient expressed a disinterest in surgery to correct the potential capsular laxity. Considering the lack of pain and the chronicity of the dysfunction, a conservative approach was recommended to the patient.

The goals of treatment were to decrease the dysfunction in her shoulder movement by improving the development of optimum motor patterns and improving muscular balance of the shoulder girdle.

Monfils, et al [21] state that "motor skill acquisition occurs through modification and organization of muscle synergies into effective movement sequences".

**Figure 4:**
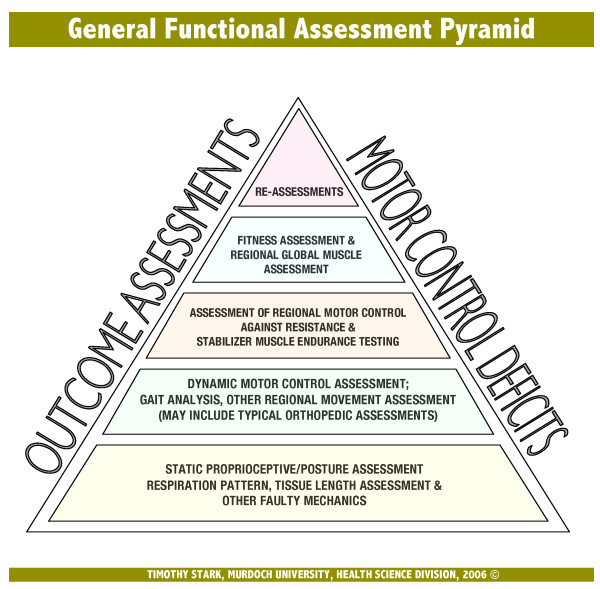
see attached pdf file named "Figure 4".

Because of the documented importance of muscle synergy and the progressions of motor control and stabilizer function [18] we decided to employ the General Functional Assessment Pyramid (Figure 4).

### Re-assessment

The patient was seen fortnightly for several weeks and was reassessed at every visit. The patient's initial trapezius complaint resolved within the third treatment. The single most important outcome measure utilised was the degree of shoulder abduction (performed while co-contracting the shoulder) obtained before dysfunction (subluxation-relocation) resulted.

Degree of left shoulder abduction before subluxation-relocation results are detailed in Table 1.

**Table 1:** 

DATE	With conscious co-contraction of the left shoulder
Day 1 (baseline)	100
2 weeks	120
4 weeks	170
6 weeks	180

At six weeks it was also observed that the patient could abduct her left shoulder to 105°C without conscious contraction of the glenohumeral and scapular stabilisers before dysfunction resulted. That was an improvement from the baseline of 75°C.

## Discussion

This patient demonstrated unusual kinesiopathology of the left shoulder (in abduction) consisting of dysrhythmia of the glenohumeral joint and early lateral rotation of the scapula. It is important in cases such as these to consider the possible anatomical and functional causes of such a disturbed motion pattern.

We chose to use an untested intervention to assist with examination and treatment. We specifically instructed the patient to cognitively (consciously) co-contract the shoulder girdle while performing an active range of motion assessment. As it transpired the patient was able to cognitively correct the dysrhythmia by co-contracting the shoulder. It is worth considering that in such patients there may be a component of neuromuscular dysfunction causing the multiple subluxation patterns.

The improvement during examination with co-contraction led the practitioners to suggest a rehabilitation protocol that was based on the patient's cognitive ability to co-contract during activities of daily living and then subsequently to progress on to stabilizer motor control exercises and strengthening.

As appreciated in the fluoroscopy video, there is apparent premature scapular movement and an obvious inability to stabilize the scapula and glenohumeral (GH) joint throughout the abduction movement. Scapular dyskinesia is common, especially with impingement disorders [22]. In order for the shoulder complex to function smoothly, the scapula must have an adequate amount of stability [17] as well as the GH joint [10]. This stability may initially require cognitive facilitation of the scapula stabilizers and the glenohumeral joint stabilizers.

Particular attention to the proximal-to-distal kinetic chain [23] may further benefit the order of stability training needed to establish an optimum motor pattern. As the patient is attempting to re-train the necessary muscle function he/she may incorporate co-contraction of the region. Co-contraction has been appreciated as a mechanism to potentially enhance joint region efficiency during moments of increased accuracy demand [13,24]. Co-contraction is a simple non-invasive manoeuvre that, as demonstrated in this case, can be implemented as an assessment element just as it can a rehabilitation element.

Although this patient's condition improved no conclusion is drawn from it. However, the assessment and rehabilitation method was implemented in a logical sequence for this case and could provide the basis of a hypothesis for testing. The background information for this clinical presentation was limited to one search engine. Further resources should be utilized if a case series is considered.

The General Functional Assessment Pyramid and the General Physical Rehabilitation Pyramid used in this case report has not been subjected to research testing. The pyramids contain many parts. It may be that some parts are effective while others are not, or indeed that the pyramids are not effective at all.

## Conclusion

This paper presented an unusual case of aberrant shoulder movement that highlights the need for careful examination and thought regarding the anatomical structures and neuro-motor patterns that may be involved or compromised. It also emphasised the use of co-contraction during examination in an attempt to immediately improve a dysrhythmia. While there are numerous treatments proposed in the literature for shoulder dysfunction, few have been held to the scrutiny of a trial [25]. We suggest that further research take place with properly conducted trials on groups of similar patients.

## Competing interests

Dr. Tim Stark is the developer of the General Functional Assessment Pyramid and the General Physical Rehabilitation Pyramid used in this case.

## Authors' contributions

All noted authors have read and approved the final manuscript.

TS is the primary author and provided literary content involving history and current trends for shoulder rehabilitation. TS also consulted on this case providing the direction of patient examination and rehabilitation.

JS provided the on-going treatment and provided literary content for the case.

BW provided guidance in drafting the manuscript.

NM provided guidance on fluoroscopy positioning and patient imaging coordination.

JC provided clinical comment on the patient's plain film radiographs and fluoroscopy and editing input.

## Supplementary Material

Additional File 1 Video 1. Gross range of motion into bilateral abduction demonstrating the aberrant glenohumeral rhythm. Click here for file

Additional File 2 Video 2. Gross range of motion into abduction; right shoulder normal, left shoulder first demonstrates the aberrant glenohumeral rhythm, the patient is instructed to co-contract the left shoulder complex resulting in an immediate near-normal abduction rhythm. Click here for file

Additional File 3 Video 3. A fluoroscopy of the involved shoulder; the first four abduction movements demonstrate the aberrant pattern specifically the early lateral rotation of the scapula and multiple subluxation tendencies of the glenohumeral joint. The following four abduction repetitions demonstrate a much smoother rhythm while the patient was co-contracting the shoulder complex. Click here for file
